# Supplementary material for: Splice-Junction-Based Mapping of Alternative Isoforms in the Human Proteome
Source: Cell Rep. Author manuscript; Available in PMC 2020 Jan 15. (PMC6961840; doi:10.1016/j.celrep.2019.11.026)
Supplement: 3 [file NIHMS1546469-supplement-3.zip › DF2/PXD0010154/Thyroid-152-P43405-IGTQGNVNFGGRPQLPGSHPASSPAQGNR.pdf]

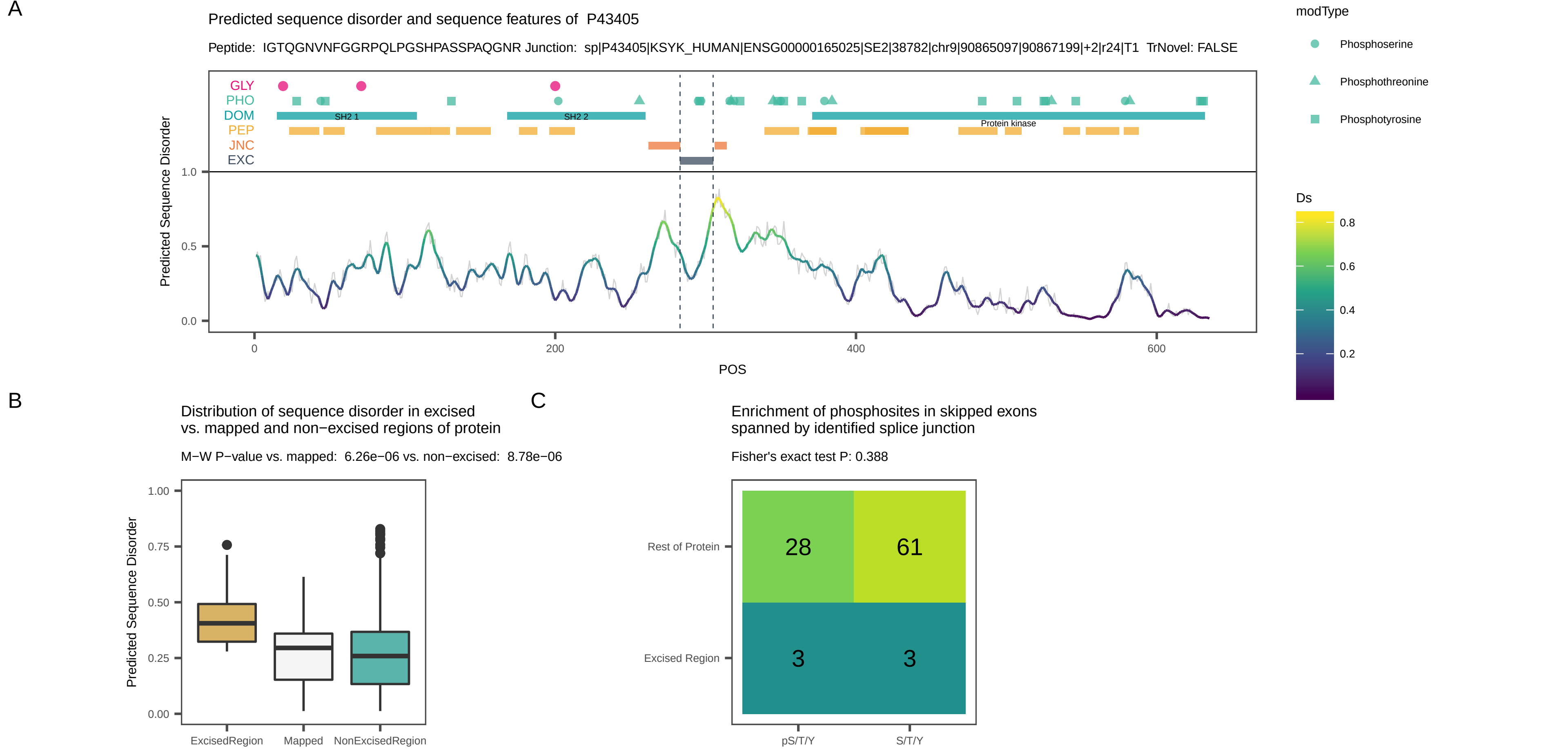

A
Predicted sequence disorder and sequence features of P43405
Peptide: IGTQGNVNFGGRPQLPGSHPASSPAQGNR Junction: sp|P43405|KSYK_HUMAN|ENSG00000165025|SE2|38782|chr9|90865097|90867199|+2|r24|T1 TrNovel: FALSE
GLY
PHO
DOM
PEP
JNC
EXC
SH2 1
SH2 2
Protein kinase
Predicted Sequence Disorder
POS
modType
Phosphoserine
Phosphothreonine
Phosphotyrosine
Ds
B
Distribution of sequence disorder in excised vs. mapped and non−excised regions of protein
M−W P−value vs. mapped: 6.26e−06 vs. non−excised: 8.78e−06
Predicted Sequence Disorder
ExcisedRegion
Mapped
NonExcisedRegion
C
Enrichment of phosphosites in skipped exons spanned by identified splice junction
Fisher's exact test P: 0.388
Rest of Protein
Excised Region
28
61
3
3
pS/T/Y
S/T/Y
